# Perspectives on Professionalism Among Postgraduate Students at JIIU’s Indian Institute of Medical Science and Research, Jalna, Maharashtra

**DOI:** 10.7759/cureus.115545

**Published:** 2026-08-31

**Authors:** Mohammad Shafee, Ilyas Bemat, Ishrath Fathima, Alka Rawekar, Tooba Fatima, Azhar Siddiqui

**Affiliations:** 1 Community Medicine, Indian Institute of Medical Science and Research, Aurangabad, IND; 2 Physiology, JIIU's Indian Institute of Medical Science and Research, Jalna, IND; 3 Obstetrics and Gynecology, JIIU's Indian Institute of Medical Science and Research, Jalna, IND; 4 Physiology, Jawaharlal Nehru Medical College, Datta Meghe Institute of Medical Science, Wardha, IND; 5 Pathology, JIIU's Indian Institute of Medical Science and Research, Jalna, IND; 6 Anatomy, JIIU's Indian Institute of Medical Science and Research, Jalna, IND

**Keywords:** altruism, duty of care, intervention, postgraduates, professionalism

## Abstract

Background: Professionalism is a very important aspect of medical practice. Every medical doctor must practice medicine with professionalism. Professionalism among postgraduates (PGs) must be inculcated so that they practice with professionalism in the future.

Aims and objectives: To assess perspectives on professionalism among PGs.

Materials and methods: An interventional study was conducted among 59 PGs studying at JIIU’s Indian Institute of Medical Science and Research, Badnapur, Jalna, in various departments from March 2022 to February 2023. Baseline data were collected using a structured proforma by conducting a Mini Clinical Examination Exercise. An intervention in the form of a two-hour lecture on professionalism was conducted. Post-intervention data were collected and analyzed using appropriate statistical tests.

Results: A total of 59 PG students participated in the study, of whom 30 were first-year junior residents, and 29 were second-year junior residents. Of these, 37 (62.71%) were males, and 22 (37.29%) were females. The mean age of these students was 27.6 years (SD of 4.06 years). The pre-test mean score was highest i.e 4.1[SD 0.4] for domain of Duty, followed by the domains of Clinical Excellence and Responsibility and Accountability. The domain of Altruism showed the lowest mean pretest score of 2.5m[SD 0.5]. The total mean pre-test score of all the domains was 3.27 (SD 0.63), which significantly increased to 4.45 (SD 0.5) in the post-test.

Discussion: Imparting knowledge on professionalism ensured improved performance among PG students with respect to all the domains of professionalism. Hence, it is highly essential to acknowledge the need to include teaching and assessing professionalism as an integral part of the medical curriculum and to implement it practically.

Conclusion: This study concluded that, teaching attributes of professionalism among medical students can significantly alter their professional behaviour.

## Introduction

Professionalism is a core competency required to practice the art of medicine [1]. In 2002, professional competence was defined by Epstein and Hundert as "the habitual and judicious use of communication, knowledge, technical skills, clinical reasoning, emotions, values, and reflection in daily practice for the benefit of the individual and community being served" [2].

In today’s era, the concept of medical professionalism is evolving from autonomy to accountability, from expert opinion to evidence-based medicine, and from self-interest to teamwork and shared responsibility.

The postgraduate (PG) medical training environment differs significantly from the undergraduate training environment, as PG students are constantly exposed to a large number of patients and shoulder more responsibilities in complex situations, both individually and as part of the departmental team [3]. Their seniors and faculty act as role models for motivation and inspiration, helping them gain and imbibe attitudes toward various attributes of professionalism, such as clinical excellence and clinical competence, humanism, accountability, altruism, ethical and legal understanding, and communication skills [1].

It has been reported in the literature that medical undergraduate and PG students who exhibit unprofessional behavior during their training period are more likely to be found guilty of professional misconduct in medical practice. Thus, it has been globally acknowledged that formal medical education should include teaching and assessment of professionalism [2].

Earlier, professionalism was a hidden curriculum in medical education. With the introduction of competency-based medical education (CBME) by the National Medical Council of India, teaching and assessing professionalism have become mandatory in both undergraduate and PG medical curricula.

Teaching and learning professionalism should start as soon as medical students enter medical school. It can be taught by various methods, such as didactic lectures, interactive sessions, role-playing, role modeling, feedback, group discussions, case-based discussions, reflection, ethical rounds, and reports [4,5]. Assessment is a driving force for learning. Professionalism can be assessed using various tools, such as multisource feedback (MSF), patient opinions, healthcare outcomes, critical incident reports, observed real or standardized patient encounters (mini-CEX (clinical evaluation exercise), PMEX (professional mini evaluation exercise), and OSCE (objective structured clinical examination)), reflective/narrative portfolios, simulation, self-assessment, case-based discussions, and paper-based assessments (multiple-choice questions (MCQ)/short-answer questions (SAQ)) [2,6,7].

Professionalism is a cornerstone of a trustworthy doctor-patient relationship, patient satisfaction, and enhanced healthcare outcomes. PG medical students are usually the first point of contact with patients in an institute, and hence, this study aimed "to assess the baseline professionalism perspectives among PG medical students and evaluate the effectiveness of a structured teaching intervention on improving their professionalism scores using mini-CEX." The seven domains assessed are clinical excellence, respect for patients' dignity, communication, duty, altruism, honor and integrity, responsibility, and accountability. The study measures both baseline and post-intervention perspectives of professionalism.

## Materials and methods

This was a pre- and post-comparison study conducted over a period of one year among PG medical students of JIIU’s Indian Institute of Medical Science and Research, Badnapur, Jalna, Maharashtra. Approval was obtained from the head of the institute and the Institutional Ethics Committee.

Other students pursuing MBBS (Bachelor of Medicine, Bachelor of Surgery) and BSc (Bachelor of Science) nursing at this institute were excluded from this study.

A total of 59 PG medical students were included in the study by purposive sampling (Figure 1). These PGs belonged to various departments: Medicine, Surgery, Obstetrics and Gynecology, Ophthalmology, Dermatology, Orthopaedics, Psychiatry, and Pathology.

**Figure 1:**
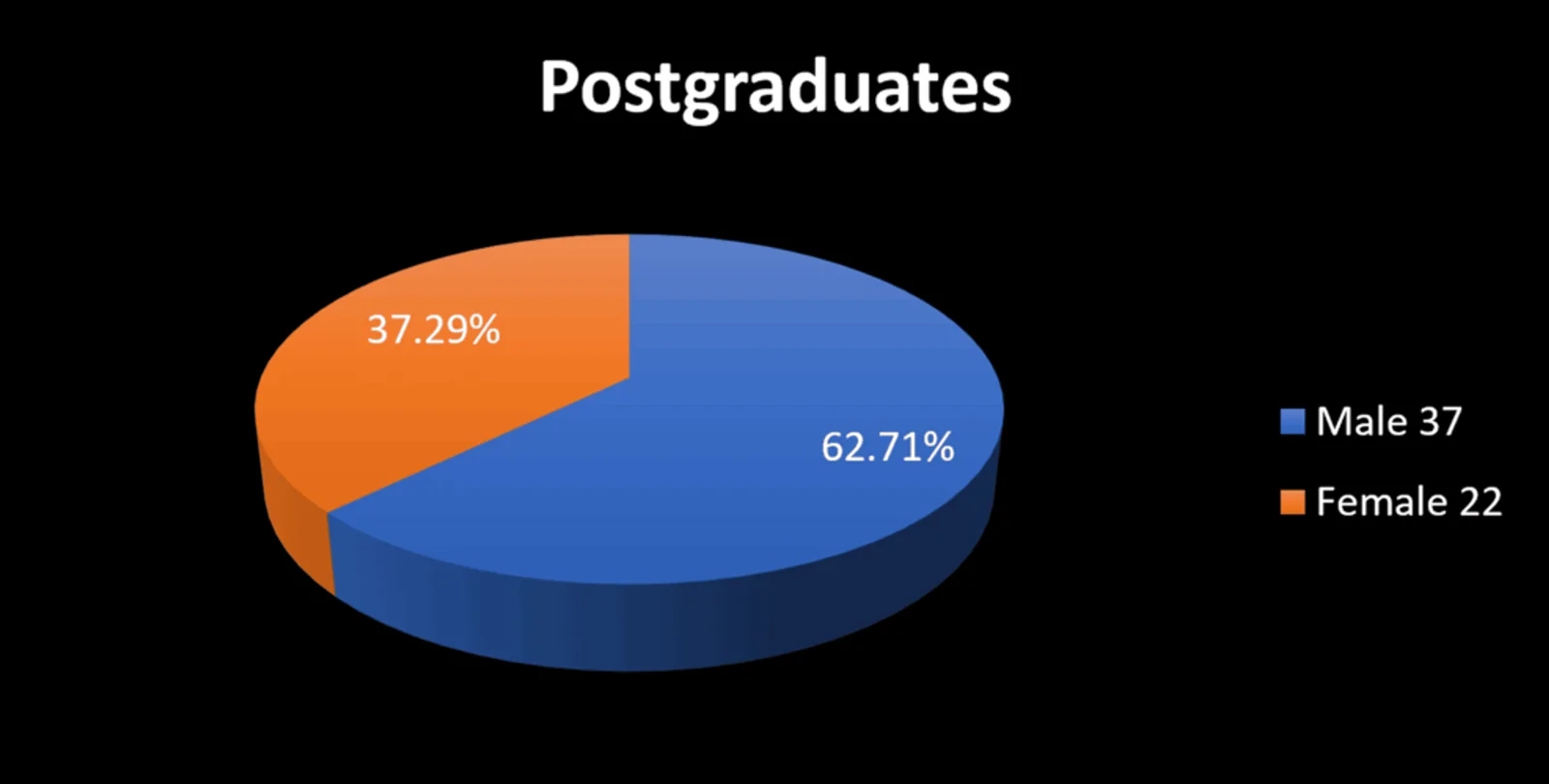
Gender-wise distribution of postgraduate students

Workplace-based assessment (WPBA) in the form of mini-CEX was planned for assessing various attributes/domains of professionalism, which included clinical excellence, respect for patients' dignity, communication, duty, altruism, honor and integrity, responsibility, and accountability.

A closed-ended questionnaire/checklist consisting of 33 items for seven domains of professionalism was developed for mini-CEX by referring to various sources from the literature (Appendix A) [8-12]. Select faculty representatives reviewed the survey questions during the development stage for face and content validity. Cronbach's alpha was calculated to estimate the internal consistency of the 33 items in our survey and was found to be 0.71. The level of professionalism in all domains was assessed by a single trained assessor (training in the form of briefing and hands-on training) using mini-CEX through direct observation in outpatient department settings with 59 different patients. Baseline data were obtained during the study period from PGs, not at the beginning of residency. Each item in the checklist was assessed on a Likert scale of agreement from 1 to 5, in which 1 indicated strongly disagree, 2 indicated disagree, 3 indicated neither agree nor disagree, 4 indicated agree, and 5 indicated strongly agree. The rating of each item was calculated from the Likert scale scores, which ranged from a minimum score of 59 (if all PGs scored 1) to a maximum score of 295 (if all PGs scored 5). The total score for each item was obtained. The mean score and SD from these total scores were obtained for each individual domain of professionalism. The mean scores ranged from 1 to 5.

Some items under the domains of duty, altruism, honor and integrity, responsibility, and accountability were scored based on feedback received from the heads of the respective departments to which the PGs belonged.

This was followed by a two-hour interactive educational awareness session conducted by the trained primary investigator regarding all domains of professionalism for all 59 PG students. The educational intervention included a presentation on seven domains of professionalism followed by an interactive session with all 59 PG students.

After this, the PGs were again assessed one by one using mini-CEX for their level of professionalism in the posttest. During this assessment, different patients were attended to by the PGs, as the previous patients were not available at this time. Mini-CEX was assessed by the same assessor.

The mean and SD of the PGs' pretest and posttest scores were compared by applying Student's paired t-test.

The PG students were sensitized about the study, and informed consent was obtained from them. This was followed by an intervention in the form of imparting knowledge about professionalism to all the PGs. Again, professionalism was assessed in the PGs post-intervention using the same WPBA tool as used before the intervention.

The data obtained were entered into Microsoft Excel (Microsoft Inc., Redmond, VA, USA) and analyzed using appropriate statistical measures, such as mean and SD. A P-value <0.05 was considered significant.

## Results

Table 1 shows pretest and posttest mean scores (Likert scale of agreement from 1 to 5) for each item/parameter and total mean scores for each domain in mini-CEX. Table 1 shows that the domain of clinical excellence had a mean pretest score of 3.56 (SD 0.51) and a mean posttest score of 4.78 (SD 0.46), and this difference between the mean pretest and posttest scores was statistically significant, with a P-value of 0.000001. In this domain, the parameter "Does complete clinical examination" shows the highest mean pretest and posttest scores. The parameter "Advices proper treatment of specific medical conditions/Performs specific procedures" shows the lowest mean pretest score but is among the parameters showing the highest posttest scores.

**Table 1:** Distribution of pretest and posttest mean scores by Mini-CEX for professionalism among postgraduates CEX, clinical evaluation exercise

Professionalism domains	Pretest mean score	Pretest SD	Posttest mean score	Posttest SD	P-value
Clinical excellence	3.56	0.51	4.78	0.46	0.000001
1. Takes complete history from patient	4.2	0.4	5	0.4	
2. Does complete clinical examination	4.5	0.5	5	0.4	
3. Draws a conclusive diagnosis	4.4	0.5	4.6	0.5	
4. Advises proper treatment of specific medical conditions / Performs specific procedures	2.55	0.5	5	0.5	
5. Provides choices to the patient as appropriate but is also willing to recommend a specific course of treatment	2.85	0.5	4.6	0.5	
6. Creates treatment plans	3.73	0.8	4.5	0.5	
7. Explains side effects of medications	2.05	0.5	5	0.4	
8. Orders tests and analyzes their results	4.2	0.4	4.6	0.5	
Respect for patients' dignity	3.22	0.85	4.5	0.45	0.0000029
1. Avoids inappropriate behavior or words to patient	3.5	1	4.5	0.5	
2. Makes eye contact with the patient	3.2	0.9	4.2	0.4	
3. Makes eye contact with the family members	3.35	1	4.4	0.5	
4. Listens carefully when the patient is describing the medical concern	2.85	0.5	4.9	0.4	
Communication	2.81	0.5	4.28	0.57	0.000001
1. Greets patient when he enters doctor's or examination room	2.4	0.5	4.5	0.5	
2. Understands and cares for patient	2.7	0.5	4.3	0.5	
3. Asks history of disease	4.5	0.5	5	0.5	
4. Explains diagnosis and prognosis	2.85	0.5	4.7	0.5	
5. Uses appropriate physical contact	2.4	0.5	4.3	0.5	
6. Is attentive and present to the patient and the situation	2.6	0.5	3.89	0.7	
7. Uses appropriate humor	2.2	0.5	3.8	0.7	
8. Acknowledges patient's family	2.85	0.5	3.8	0.7	
Duty	4.1	0.4	4.8	0.44	0.000001
1. Ready to perform duty when it is scheduled	4.2	0.4	5	0.4	
2. Reports to the duty on time	4.08	0.4	4.8	0.4	
3. Available during duty time	4.12	0.4	4.7	0.5	
4. Does not remain absent without prior permission	4.2	0.4	4.7	0.5	
5. Indicates willingness to spend adequate time with patient through unhurried movements	3.9	0.4	4.8	0.4	
Altruism	2.5	0.5	3.87	0.55	0.000001
1. Selflessness in caring for patients	2.8	0.5	4	0.4	
2. Available for caring the patient during emergency	2.2	0.5	3.75	0.7	
Honor and integrity	3.23	0.76	4.46	0.53	0.006835
1. Doesn't make inappropriate demands	3.3	1	4.5	0.5	
2. Is not abusive and critical in times of stress	2.8	0.5	4.3	0.6	
3. Appropriately grooms and dresses while working in a professional environment	3.6	0.8	4.6	0.5	
Responsibility and accountability	3.53	0.9	4.5	0.53	0.00008587
1. Accepts blame for failure	3.5	1	4.3	0.6	
2. Follows through on tasks	3.8	0.7	4.5	0.5	
3. Arises on time	3.3	1	4.7	0.5	

The domain of respect for patients' dignity had a mean pretest score of 3.22 (SD 0.85) and a mean posttest score of 4.5 (SD 0.45), and this difference was statistically significant, with a P-value of 0.0000029. In this domain, the parameter "Asks history of disease" shows the highest mean pretest and posttest scores. The parameter "Listens carefully when the patient is describing the medical concern" shows the lowest mean pretest score but shows the highest posttest score in the domain. The parameter "Makes eye contact with the patient" has the lowest mean posttest score.

In the domain of communication, the mean pretest score was 2.81 (SD 0.5), and the mean posttest score was 4.28 (SD 0.57), and this difference between the two scores was statistically significant, with a P-value of 0.000001. In this domain, the parameter ‘Avoids inappropriate behavior or words to patient’ shows the highest mean pretest and posttest scores. The parameter "Uses appropriate humor" shows the lowest mean pretest score, which improved in the posttest but is still among the lowest posttest mean scores in this domain.

In the domain of duty, the mean pretest score was 4.1 (SD 0.4), and the mean posttest score was 4.8 (SD 0.44), and this difference was statistically significant, with a P-value of 0.000001. In this domain, the parameters "Ready to perform duty when it is scheduled" and "Does not remain absent without prior permission" show high mean pretest and posttest scores, with the highest being for "Reports to the duty on time." The parameter "Indicates willingness to spend adequate time with patient through unhurried movements" shows the lowest mean pretest score, which improved in the posttest and is among the highest mean posttest scores in this domain.

The domain of altruism shows a mean pretest score of 2.5 (SD 0.5) and a mean posttest score of 3.87 (SD 0.55), and this difference between the pretest and posttest scores was statistically significant, with a P-value of 0.000001. In this domain, the parameter "Selflessness in caring for patients" shows high mean pretest and posttest scores.

The domain of honor and integrity shows a mean pretest score of 3.23 (SD 0.76) and a mean posttest score of 4.46 (SD 0.53), and this difference between the pretest and posttest scores was statistically significant, with a P-value of 0.006835. In this domain, the parameter "Appropriately grooms and dresses while working in a professional environment" shows high mean pretest and posttest scores. The parameter "Is not abusive and critical in times of stress" shows the lowest mean pretest score, which improved in the posttest but is still among the lowest mean posttest scores in this domain.

In the domain of responsibility and accountability, the mean pretest score was 3.53 (SD 0.9) and the mean posttest score was 4.5 (SD 0.53), and this difference between the two scores was statistically significant, with a P-value of 0.00008587. In this domain, the parameter "Follows through on tasks" shows the highest mean pretest score. The parameter "Arises on time" shows the lowest mean pretest score but shows the highest mean posttest score in this domain.

Overall, the domain of duty shows the best pretest mean score, followed by the domains of clinical excellence and responsibility and accountability. Overall, the parameters "Does complete clinical examination," "Draws a conclusive diagnosis," and "Ask history of disease" show the best mean pretest scores. Whereas, the domain of altruism shows the lowest mean pretest score, followed by the domain of communication. The parameters "Explain side effects of medications," "Uses appropriate humor," and "Available for caring the patient during emergency" show the lowest mean pretest scores.

Overall, the best posttest mean score was seen in the domain of duty, followed by the domains of clinical excellence, respect for patients' dignity, and responsibility and accountability. The parameters "Takes complete history from patient," "Does complete clinical examination," and "Explain side effects of medications" show the highest mean posttest scores. Whereas, "Available for caring the patient during emergency" is the parameter with the lowest mean posttest score.

Table 2 shows a statistically highly significant (P-value=0.0000001) improvement in all domains of professionalism among PG students after the educational intervention, as shown by mini-CEX scores. The total mean pretest score of all the domains was 3.27 (SD 0.63), which significantly increased to 4.45 (SD 0.5) in the posttest.

**Table 2:** Distribution of total pretest and posttest mean scores and SDs of postgraduates on professionalism

Postgraduates	Mean score	SD	P-value
Pretest	3.27	0.63	0.000001
Posttest	4.45	0.5	

## Discussion

The virtues that define professionalism can be assessed in medical graduates and PG students by applying WPBA tools like Mini-CEX and P-MEX.

This study was designed to assess professionalism in PG medical students by applying Mini-CEX before and after an educational intervention. A total of 59 PG medical students were included in this study, consisting of 37 (62.71%) males and 22 (37.29%) females, with a mean age of 27.6 years (SD 4.06 years).

Before the educational session, the domains of duty, clinical excellence, and responsibility and accountability showed the best pretest mean scores. In these domains, the parameters that showed the best mean scores were "Draws a conclusive diagnosis," "Asks history of disease," "Does not remain absent without prior permission," "Does complete clinical examination," and "Follows through on tasks." Thus, the PG medical students revealed several qualities of disciplined clinicians even before the educational session.

However, before the instructive session, they showed poor performance with regard to the domains of altruism and communication. The mean pretest scores were low for parameters such as "Available for caring the patient during emergency," "Uses appropriate humor," "Is not abusive and critical in times of stress," "Selflessness in caring for patients," "Indicates willingness to spend adequate time with patient through unhurried movements," "Listens carefully when the patient is describing the medical concern," and "Explain side effects of medications." This suggests a lack of qualities such as responsible behavior, selflessness, interactive skills, patience, and composure among young PG medical students, which are crucial elements contributing to a lack of trust and confidence among patients toward medical professionals.

In a study by A. Razzak [10], in which P-MEX was used to assess professionalism in 32 pediatric PG students, items such as "maintained appropriate boundaries in doctor-patient relationship" and "maintained appropriate appearance" were given the highest scores.

Yali Wu et al. [13] used Mini-CEX to study the level of professionalism among PG and resident doctors and found that the overall performance score was higher among PG students than resident doctors, with better scores in the domains of history taking, physical examination, clinical diagnosis/treatment regimen, knowledge examination, communication skills, and humanistic care.

Colleen Gillespie et al. [14] conducted a survey among 85 senior residents in emergency medicine, internal medicine, pediatrics, psychiatry, and surgery specialties to assess specific behaviors reflecting professionalism. These residents reported feeling most competent in being accountable and demonstrating respect, but some residents reported having trouble being sensitive to patients, and the most frequently witnessed behavior was disrespectful behavior in the learning environment.

Rasul S et al. [15] found that honor/integrity was the most valued trait and excellence/autonomy was the lowest-scored domain of medical professionalism in a cross-sectional survey conducted among medical students from the first year, final year, and faculty members, with a significant difference between the final-year students and faculty.

In the present study, it was observed that after the teaching session, all mean scores improved significantly, especially for the domains of duty, clinical excellence, and respect for patients' dignity. The parameters that showed the most improvement were "Reports to the duty on time," "Indicates willingness to spend adequate time with patient through unhurried movements," "Advices proper treatment of specific medical conditions / Performs specific procedures," "Explain side effects of medications," and all the parameters in the domains of altruism and communication.

Thus, imparting knowledge on professionalism ensured improved performance among PG students with respect to all the domains of professionalism.

Arielle S. Berger et al. [3], in a systematic review, showed that 20 out of 35 studies reported statistically significant positive effects of teaching professionalism in PG medical students, with the most effective teaching interventions being those targeting improvements in knowledge. This review also stated that curriculum duration was not associated with effectiveness and that even simple, short teaching sessions can affect professionalism.

The strengths of the study include a highly relevant issue of professionalism in doctors, which is a much-needed trait among doctors today. Using Mini-CEX in a real outpatient department setting gives it important practical value. The results show a clear, statistically significant increase in scores after the lecture, which supports the idea that teaching matters. The study also covered seven broad domains, which gives an overall picture.

Limitations

The limitations of this study include the small sample size, the absence of a control group, the use of different patients in the posttest, the lack of comparison of professionalism between junior resident 1 and junior resident 2, and the fact that 360-degree feedback, considered the best method for assessing professionalism, was not applied in this study.

## Conclusions

In conclusion, this study indicates that a lack of professional behavior among PG medical students can be positively modified by instructive or teaching sessions about the attributes and domains of medical professionalism. Thus, teaching and periodically assessing professional behavior among medical students at all levels, after formative feedback, must become an integral part of the curriculum and must be actively implemented in all medical institutes so that future young doctors are much better aware of their professional roles. However, meticulous planning, faculty sensitization, and training will be required for its successful execution.

## Appendices

Appendix A

**Table 3:** Questionnaire for assessing professionalism among postgraduate medical students using the Mini-CEX CEX, clinical evaluation exercise

Domains of professionalism	Strongly disagree 1	Disagree 2	Neither agree or disagree 3	Agree 4	Strongly agree 5
Clinical excellence					
1. Takes complete history from patient					
2. Does complete clinical examination					
3. Draws a conclusive diagnosis					
4. Advises proper treatment of specific medical conditions/performs specific procedures					
5. Provides choices to the patient as appropriate but is also willing to recommend a specific course of treatment					
6. Creates treatment plans					
7. Explains side effects of medications					
8. Orders tests and analyzes their results					
Respect to patients’ dignity					
1. Avoids inappropriate behavior or words toward the patient					
2. Makes eye contact with the patient					
3. Makes eye contact with the family members					
4. Listens carefully when the patient is describing the medical concern					
Communication					
1. Greets patient when he enters doctor’s or examination room					
2. Understands and cares for patient					
3. Asks history of disease					
4. Explains diagnosis and prognosis					
5. Uses appropriate physical contact					
6. Is attentive and present to the patient and the situation					
7. Uses appropriate humor					
8. Acknowledges patient's family					
Duty					
1. Ready to perform duty when it is scheduled					
2. Reports to the duty on time					
3. Available during duty time					
4. Does not remain absent without prior permission					
5. Indicates willingness to spend adequate time with patient through unhurried movements					
Altruism					
1. Selflessness in caring for patients					
2. Available for caring the patient during emergency					
Honor and integrity					
1. Doesn’t make inappropriate demands					
2. Is not abusive and critical in times of stress					
3. Appropriately grooms and dresses while working in a professional environment					
Responsibility and accountability					
1. Accepts blame for failure					
2. Follows through on tasks					
3. Arises on time					
